# Species Richness and Trophic Diversity Increase Decomposition in a Co-Evolved Food Web

**DOI:** 10.1371/journal.pone.0020672

**Published:** 2011-06-03

**Authors:** Benjamin Baiser, Roxanne S. Ardeshiri, Aaron M. Ellison

**Affiliations:** 1 Harvard Forest, Harvard University, Petersham, Massachusetts, United States of America; 2 Environmental Science, Policy and Management, University of California, Berkeley, California, United States of America; National Institute of Water & Atmospheric Research, New Zealand

## Abstract

Ecological communities show great variation in species richness, composition and food web structure across similar and diverse ecosystems. Knowledge of how this biodiversity relates to ecosystem functioning is important for understanding the maintenance of diversity and the potential effects of species losses and gains on ecosystems. While research often focuses on how variation in species richness influences ecosystem processes, assessing species richness in a food web context can provide further insight into the relationship between diversity and ecosystem functioning and elucidate potential mechanisms underpinning this relationship. Here, we assessed how species richness and trophic diversity affect decomposition rates in a complete aquatic food web: the five trophic level web that occurs within water-filled leaves of the northern pitcher plant, *Sarracenia purpurea*. We identified a trophic cascade in which top-predators — larvae of the pitcher-plant mosquito — indirectly increased bacterial decomposition by preying on bactivorous protozoa. Our data also revealed a facultative relationship in which larvae of the pitcher-plant midge increased bacterial decomposition by shredding detritus. These important interactions occur only in food webs with high trophic diversity, which in turn only occur in food webs with high species richness. We show that species richness and trophic diversity underlie strong linkages between food web structure and dynamics that influence ecosystem functioning. The importance of trophic diversity and species interactions in determining how biodiversity relates to ecosystem functioning suggests that simply focusing on species richness does not give a complete picture as to how ecosystems may change with the loss or gain of species.

## Introduction

Understanding the causes and consequences of biodiversity is a central focus of ecological research. Variation in species richness, composition, and network structure has implications for population dynamics [1]–[3], resistance to species invasions [4], [5], and a suite of ecosystem functions including decomposition [6], [7], primary productivity [8], [9], seed dispersal [10], and pollination [11]. Knowledge of how the number of species relates to ecosystem functioning is important for understanding the maintenance of diversity and the potential effects of species losses or gains on ecosystems [12]–[14]. However, not all species are equal. As ecological communities vary in species richness and composition, they also vary in traits, types of ecological interactions, and food web structure. Recent advances in methods for incorporating traits and trophic position (*i.e.* the prey and predators of a given species) into diversity measurements [15]–[20] provide an assessment of how these important components of biodiversity influence ecosystem functioning. Considering species traits and food web structure in addition to species richness may also reveal mechanisms by which diversity relates to ecosystem functioning.

Here, we use a model system, the aquatic food web that inhabits the water-filled leaves of the northern pitcher plant, *Sarracenia purpurea* [21]–[24], to explore the relationship between decomposition rate (an indicator of the rate of mineralization of organic matter) and both species richness and trophic diversity (TD), a trophic-based measure of functional diversity [17], [25], [26], in a complete food web. The *Sarracenia* food web is an ideal system; it is a complete, 5-trophic level web in which four of the species have a shared evolutionary history with the pitcher plant and require this habitat to complete their life cycles [27]. As a result, our experimental food webs reflect realistic diversity levels and assembly (or disassembly) scenarios [28]−[30] rather than random assemblages; nearly all experimental species combinations have been observed in naturally occurring pitchers sampled across the range of *Sarracenia purpurea* [23], [31].

We conducted a greenhouse experiment in which we assembled 70 food webs ranging from 0 consumer species (*i.e.* containing bacteria only) to the full complement of 9 consumer species (Table S1). For each species richness treatment, we divided the range of TD into seven equally-spaced levels from the lowest to the highest possible TD values. Each food web was assembled in an individual pitcher of an individual *Sarracenia* plant. To explore the influence of species richness and TD on decomposition, we measured the mass-loss of one carpenter ant (*Camponotus pennsylvanicus*) in each pitcher over a two-week period. Ants are the primary prey of pitcher plants, accounting for >75% of the prey captured [32].

There are three direct pathways of decomposition in the *Sarracenia* food web, two of which we explored in our experiment. The first is bacterial decomposition, which represents the majority of decomposition in the food web [24], [33]. The second is shredding of prey carcasses into particulate organic matter by the midge (*Metriocnemus knabi*) which also facilitates bacterial decomposition [34], [35]. The sarcophagid fly (*Fletcherimyia fletcheri*) is also a shredder in the *Sarracenia* food web, but it is relatively uncommon across the range of *S. purpurea* [31] and was not used in this experiment. Indirect pathways include effects of consumers on bacterial density and composition [33], [36], [37], which in turn influence bacterial decomposition.

**Figure 1:**
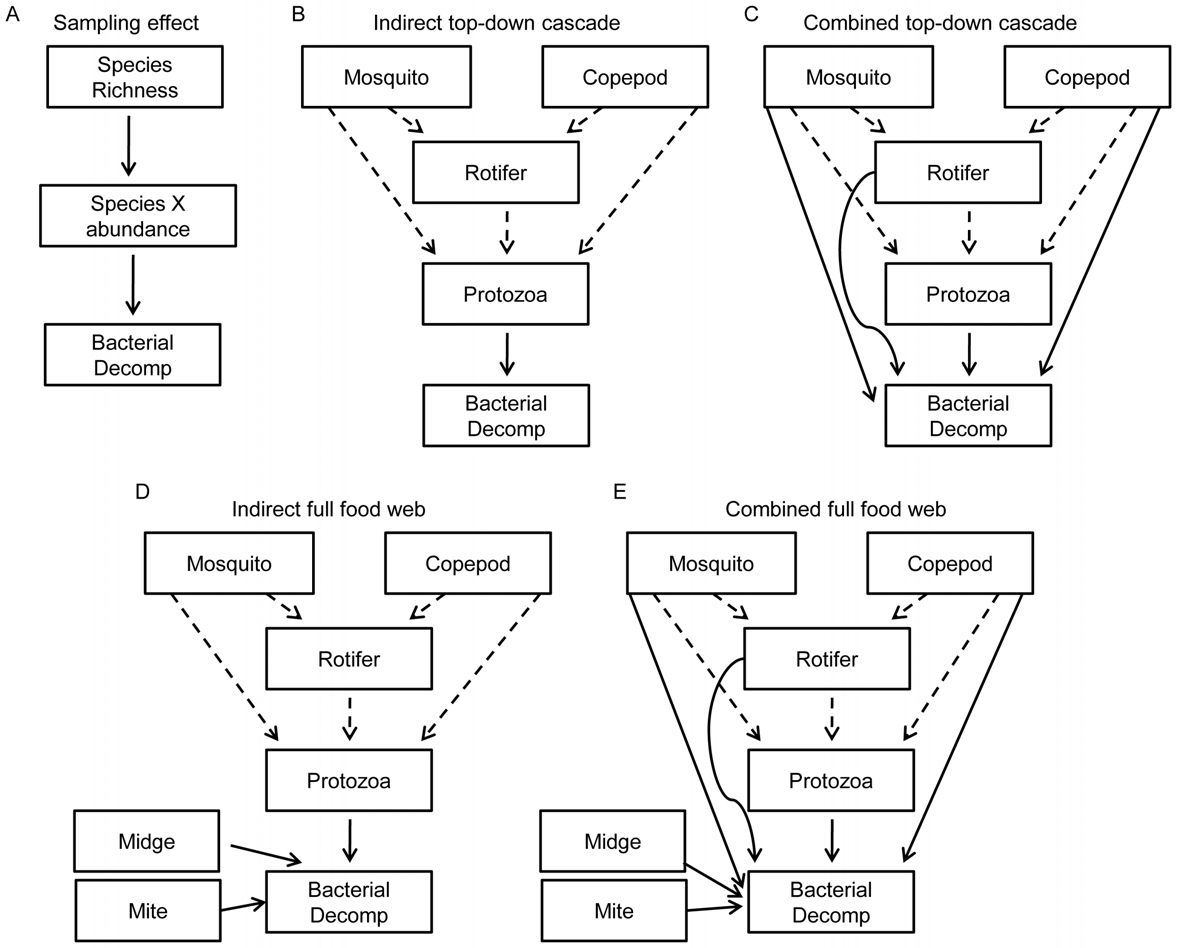
Path models ( =  *a priori* hypotheses) describing decomposition dynamics in the *Sarracenia* food web. (**A**) The *sampling effect* model tests if high species richness increases the probability that an ‘important’ decomposer will be present. This model was run for each species to determine if any one species contributes disproportionately to decomposition. (**B**) The *indirect top-down cascade* model tests if mosquitoes, copepods, and rotifers indirectly facilitate bacterial decomposition by releasing bacteria from predation by protozoa (dashed lines). (**C**) The *combined top-down cascade* model tests if the indirect effects (dashed lines) from mosquitoes, copepods, and rotifers along with direct predation (solid lines) on bacteria alter decomposition. The *indirect* (**D**) and *combined* (**E**) *full food web* models include paths for predation by the mite (*Sarraceniopus gibsoni*) on bacteria and the direct decomposition by the midge (*Metriocnemus knabi*) as pathways that influence decomposition in addition to the pathways in models (**B**) and (**C**) respectively.

We first used linear regression to test the relationship between species richness, TD, and decomposition and further investigated these relationships by using path analysis to test directly the two mechanisms that have been proposed to explain the relationship between biodiversity and ecosystem functioning: the sampling effect [28], [29] and niche complementarity [8], [38]. We constructed path models for each hypothesis and assessed the fit of each path model to the experimental data. *Sampling effect* path models simply identify relationships between species richness and specific species that contribute disproportionately to decomposition (Fig. 1a). We constructed one sampling effect path model for each species in the experiment to determine if any one species was responsible for the majority of the decomposition.

*Food web* path models represent more precisely the niche complementarity hypothesis. For the *Sarracenia* food web, we wanted to test if known trophic interactions, which represent different trophic strategies or niches, influenced decomposition. The top predator in this system — larvae of the pitcher plant mosquito, *Wyeomyia smithii* — has an indirect positive effect on bacterial abundance by reducing the abundance of protozoan and rotifer consumers [33], [36], [37]. To test if indirect pathways for the mosquito, rotifer, and an unidentified copepod increased decomposition we built the *indirect top-down trophic cascade* model (Fig. 1b). Trophic cascades have been shown to alter ecosystem functioning and population dynamics across a wide variety of systems including the *Sarracenia* food web [33], [36]–[39]. To account for mosquito, copepod, and rotifer predation on bacteria, we built a *combined top-down trophic cascade* path model that also includes direct paths from these consumers to bacteria as well as the indirect paths (Fig. 1c).

While top-down effects of *Wyeomyia smithii* have been shown to independently alter bacterial abundance and composition [33], [36], shredding of detritus by the midge and foraging by the histiostomatid mite, *Sarraceniopus gibsoni* [40], [41], interact with top-down effects and alter population dynamics in the *Sarracenia* food web [34]–[37]. We built two models that incorporate the midge and mite along with top-down dynamics. The *indirect full food web* model (Fig. 1d) includes the indirect top-down trophic cascade pathways controlling bacterial decomposition, direct predation on bacteria by the mite [40], [41] that may decrease bacterial abundance or activity, and direct decomposition by the midge. The *combined full food web* model (Fig. 1e) includes the mite and midge pathways along with the direct and indirect top-down paths. Finally, we also assessed the influence of prey input (*i.e.* initial ant mass) on decomposition in these models.

## Results

**Figure 2:**
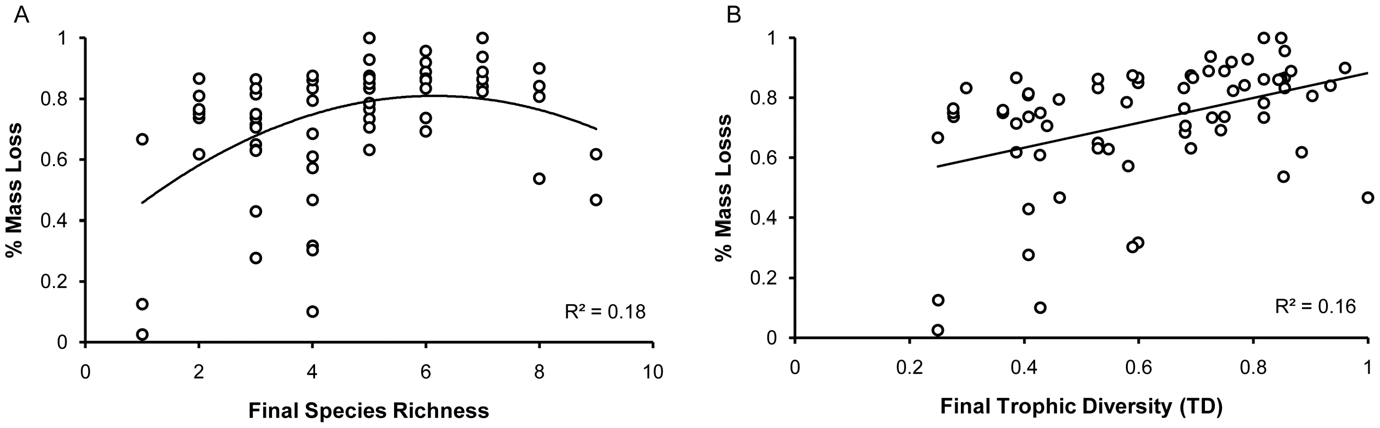
Relationship between final species richness, final trophic diversity (TD), and decomposition. Decomposition showed a ‘hump-shaped’, polynomial relationship with species richness (**A**) and a positive linear relationship with TD (**B**). Regression models (lm in R v.2.11.1) are significant at P<0.05.

Decomposition showed a ‘hump-shaped’, polynomial relationship with species richness (F_2,67_ = 8.3, *P* = 0.0006; Fig. 2a) and a positive linear relationship with TD (F_1,68_ = 14.59, *P* = 0.0003; Fig. 2b). Species richness and TD were highly correlated (*R* ^2^ = 0.82, F_1,68_ = 306.4, *P*<0.0001; Figure S1). On average mass loss in ants across treatments was 72% (N = 70, SD = 21%). Ant decomposition data is available at the Harvard Forest data archive (http://harvardforest.fas. harvard.edu/data/archive.html) data set HF-169.

**Figure 3:**
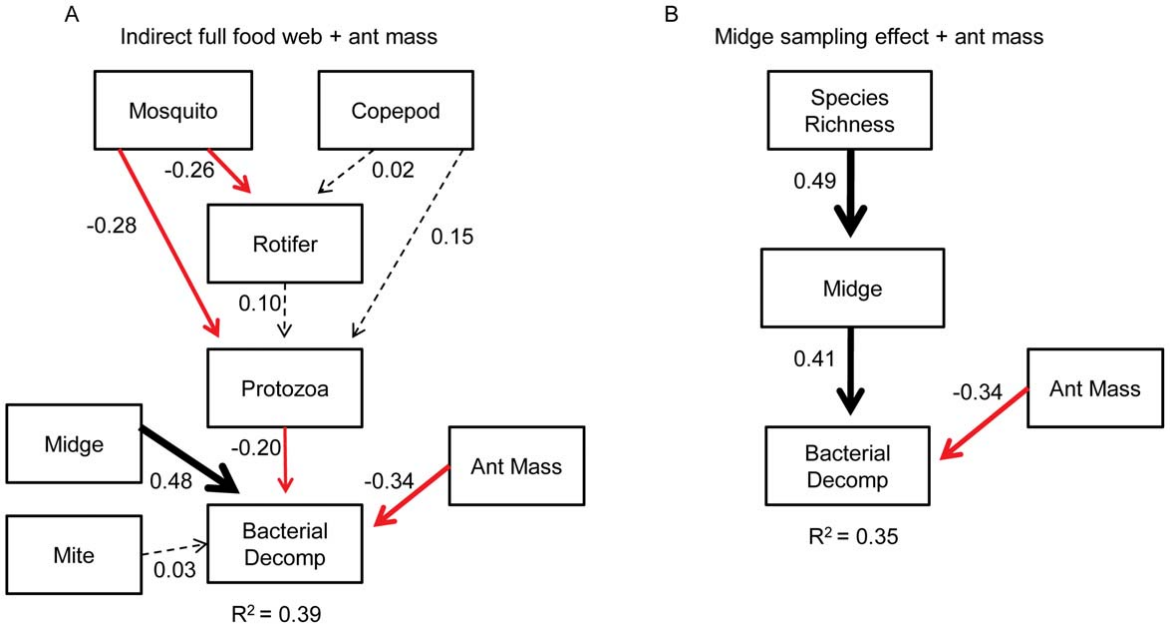
Best-fit path models describing decomposition dynamics in the *Sarracenia* food web. The *indirect full food web* model including ant mass (**A**) was the top overall model and the *midge sampling effect* model including ant mass (**B**) was the top sampling effect path model explaining decomposition by bacteria (Decomp) in the *Sarracenia* food web. Standardized path coefficients are shown for each link and arrows are proportional to the magnitude of the coefficient. Solid black lines indicate significant (*P*<0.05) positive relationships and solid red lines indicate significant negative relationships. Dashed lines indicate non-significant relationships. The R^2^ value shows the amount of variation in decomposition explained by the path model.

Sampling effect and food web path models were used to further explore how species richness and TD increase decomposition. The top ranked model was the *indirect full food web* model which also included ant mass (Fig. 3a, Table 1). This model revealed three significant pathways (regression coefficients *P*<0.05) and explained 39% of the variation in decomposition. The first pathway was a trophic cascade in which mosquito larvae had a negative effect on protozoan abundance, which in turn had a negative effect on decomposition by bacteria. Although we did not measure bacterial abundance directly, a trophic cascade induced by mosquito predation has been demonstrated repeatedly in the *Sarracenia* food web [33], [36], [37]. The copepod, which has the same prey as the mosquito larvae, had no significant influence on prey items or decomposition (Fig. 3a).

**Table 1:** Best-fit *food web* and *sampling effect* path models for decomposition in the *Sarracenia* food web.

Model	Paths	Chi-Square^1^	DF	*P*	CFI^2^	BIC^3^	Δ BIC	R^2^
Food web	Indirect full web + ant mass*	17.78	19	0.54	1	−62.94	0	0.39
Food web	Combined full web + ant mass	17.23	16	0.37	0.97	−50.75	12.19	0.39
Food web	Indirect full web	14.62	13	0.33	0.95	−40.61	22.33	0.29
Food web	Indirect top-down + ant mass	3.57	8	0.89	1	−30.42	32.52	0.18
Food web	Combined full web	13.784	10	0.18	0.88	−28.7	34.24	0.29
Food web	Combined top-down + ant mass	1.62	5	0.9	1	−19.63	43.31	0.19
Food web	Indirect top-down	3.08	4	0.55	1	−13.92	49.02	0.05
Sampling effect	midge + ant mass*	1.96	3	0.58	1	−10.79	52.15	0.35
Food web	Combined top-down	0.03	1	0.87	1	−4.22	58.72	0.09
Sampling effect	midge	1.73	1	0.19	0.98	−2.52	60.42	0.25

1 Chi-square test (a *P*-value >0.05 indicates a good fit) and the

2 Comparative Fit Index (CFI; ref. 73) >0.95 indicates a good fit between the hypothesized model and the observed data (74).

3 We compared models using the Bayesian Inference Criterion (BIC; ref. 75) and considered model(s) with ΔBIC <6 as having strong support (76).

*Path model shown in Figure 3.

The second pathway was the direct positive effect of midge larvae on decomposition; this pathway also was identified in the *midge sampling effect* model (Table 1, Fig. 3b). Midges shred prey into particulate organic matter, reducing prey biomass and facilitating its decomposition by bacteria [34]; bacteria in turn are primarily responsible for mineralizing organic matter in the pitcher plant food web [24], [33]. The third pathway was the negative effect of ant mass on decomposition. This relationship suggests that larger ants are more difficult for midges to shred and for bacteria to decompose, or that the percent of decomposable (*i.e.* non-chitinous) mass is greater in smaller individuals. The negative effect of ant mass was significant (*P*<0.05) in all path models in which it was included. Additionally, mosquito larvae, the top-level predators in the *Sarracenia* food web, had a negative effect on rotifer abundance in all food web path models (e.g. Fig 3a). However, this negative effect did not propagate through the web and influence decomposition.

The top sampling effect model was the *midge sampling effect* model (Table 1), which revealed a significant (*P*<0.05) positive effect of species richness on midge abundance, which in turn increased decomposition (Fig. 3b). The sampling effect model for the midge identified the midge as a key component of decomposition in the *Sarracenia* food web, which was confirmed by the top food web model (Fig. 3a). Overall, the food web models were a better fit to the variance-covariance structure of the data than the sampling effect models (Table 1).

## Discussion

Our results highlight the complexity and importance of assessing relationships between biodiversity and ecosystem functioning in a multi-trophic context [42], [43]. By explicitly incorporating TD into our experimental framework, we have shown that greater species richness increases the probability that trophic niches important to decomposition are filled. Specifically, the niches of top predator and detritus shredder were identified as key trophic positions for decomposition. Furthermore, we showed that species richness and TD underlie strong linkages between food web structure and dynamics; these linkages influence decomposition rate in the *Sarracenia* food web. Decomposition by bacteria is facilitated by the shredding midge and the trophic cascade initiated by the mosquito. Facilitation and trophic cascades are well-known, important parts of the *Sarracenia* system [33], [35]–[37], and are specific examples of a broad class of facultative and predatory interactions that control ecosystem process in a wide variety of ecological systems [42]–[46]. They also provide a straightforward example of how the number of species, the traits they possess, and the resulting interactions can influence ecosystem functioning.

In the case of the *Sarracenia* food web, species identity is closely related to trophic function due to the unique evolutionary history of the species pool of this container ecosystem. Consequently, species richness is closely related to TD. The degree to which this is true for other food webs will depend largely on the trophic redundancy of the species pools from which individual webs are assembled. In the case of highly redundant species pools, variation in composition or species richness among food webs will not necessarily exclude a trophic niche from being filled [47]. However, even in the low redundancy *Sarracenia* species pool, where two species inhabit the same trophic niche (mosquito larvae and copepods) (e.g. Fig. 1c) they exert very different influences on web dynamics and decomposition (Fig. 3a). It is likely that both high trophic diversity and trophic redundancy are necessary to maintain ecosystem functioning in most ecosystems.

Our results also show that TD underpins important species interactions that are essential to ecosystem functioning. The importance of species interactions in determining how ecosystems respond to global change [22], [48]–[52] leaves open the possibility that ecosystem functioning can be altered even when species richness is conserved due to changes in trait distributions and food web structure that alter ecological interactions. The fact that species richness is increasing (due to invasions and novel assembly processes) or remaining unchanged for many taxonomic groups at local –to– regional scales [53]–[55] where ecological interactions take place, suggests that understanding how the distribution of traits and ecological interactions (e.g. predation, facilitation, parasitism, and competition) are changing may be more important than simply looking at species richness.

## Materials and Methods

### The *Sarracenia* food web

*Sarracenia purpurea* is a long-lived perennial carnivorous plant with tubular leaves that open during the growing season and fill with 5 to 50 ml of rainwater. Once open, the leaves capture invertebrate prey that serves as the base of a fully-functional “brown” food web containing bacteria, protozoa, insect larval stages, and other aquatic invertebrates [21]–[24]. The *Sarracenia* food web decomposes captured prey and releases essential nutrients to the plant [24].

We collected pitcher-plant protozoa, copepods, mites, and dipteran larvae from pitcher plants growing in the Tom Swamp Bog [56], just north of Harvard Pond in central Massachusetts (42°30′ N, 72°11′ W). Sampling was approved by the Harvard Forest research committee. Protozoa were cultured with filtered pitcher fluid (see **Food web assembly**, below) in 50-ml screw-capped jars. The rotifer *Habrotrocha rosa* Donner was cultured with tap water and baker's yeast in 50-ml screw-capped jars. Macro-invertebrates were held in 50-ml screw-capped jars with protozoa and bacteria as food sources. We rinsed each macro-invertebrate with filtered pitcher fluid before placing them into food webs to remove any “hitch-hiking” protozoa.

### Experimental design

We assessed decomposition of ant prey in *Sarracenia* food webs of varying species richness and TD. We assembled 10 species richness treatments (0–9 consumer species with the 0 level including bacteria only; Table S1) with seven replicates each for a total of 70 food webs. We constructed interaction matrices and calculated trophic diversity (TD) for every combination of species at each species richness level (see **Calculating trophic diversity (TD)**, below). We divided the range of TD equally into seven levels from low, in which species have similar trophic niches, to high, in which species have unique trophic niches, for each species richness treatment and randomly selected food webs with each of these levels in order to determine the community composition for the seven replicates in each species richness treatment. We assembled each food web into an individual leaf on a separate plant (*i.e.* 70 plants). We selected the newest leaf on each plant for food web assembly and flushed each leaf with distilled-deionized water prior to assembly.

### Food web assembly

We collected pitcher-plant fluid from the field and filtered out all particulates and protozoa >1.2 µm using a syringe filter (Arcodisc 32, 1.2 µm mesh). The remaining fluid contained only bacteria and was mixed to ensure homogeneous bacterial assemblages in all pitchers. We cultured pitcher-plant yeast in petri dishes containing 15 g of agar and 1 g of yeast extract in 1 L of tap water and re-suspended yeast in the homogenized pitcher fluid. We inoculated each pitcher with 5 ml of pitcher fluid and 0.1 ml of protozoan cultures for the webs that contained protozoa. After one week, we added an additional 5 ml of pitcher fluid, 0.1 ml of rotifers, and required macro-invertebrate species to food webs. The total volume of pitcher fluid (10 ml) and the abundances of macro-invertebrates were based on averaged empirical data for pitcher plant food webs from which the samples were collected (Table S1). We plugged pitchers with cotton and marked the 10-mL line on each pitcher to control for evaporation. We checked water levels every two days and refilled pitchers to the 10-ml line with autoclaved well water.

### Measuring ant decomposition

We collected carpenter ant workers (*Camponotus pennsylvanicus*) from a single nest to use as prey items for the experiment. We froze and then weighed each ant (±1 µg) and randomly assigned one to each pitcher. We placed ants into pitchers on the same day that macro-invertebrates were added to the food web and removed the decomposed ants and ant parts two weeks later. We air-dried the decomposed ants for two days and then re-weighed them (±1 µg). We measured decomposition as percent ant mass lost over the two-week time period. To ensure that mass loss was not due to submersion in fluid and/or desiccation, we followed the same methods, using 10 carpenter ants submerged in glass jars filled with 10 ml of distilled-deionized water, placed next to plants in the greenhouse as a control. Decomposition of control ants in distilled-deionized water was significantly lower than decomposition of ants in food web treatments (two-sample *t* test: *t* = −5.5, df = 78, *P*<0.0001). A control containing only pitcher enzymes and water was not conducted because unlike other North American pitcher plants, *Sarracenia purpurea* releases low levels of enzymes [57], [58] that have little effect on decomposition and it is not possible to remove bacteria from the pitcher solution. Thus, our zero species (*i.e.* bacteria only) treatments are in essence a control absent of eukaryotic organisms.

### Calculating trophic diversity (TD)

We constructed an interaction matrix (Table S2) based on our observations and on published accounts of trophic interactions among members of the *Sarracenia* food web [21], [22], [24], [40], [41], [59], [60]. Each element *a_ij_* of this interaction matrix equals 1 if the species in row *i* consumes or is consumed by the species in column *j*, and equals 0 if no trophic interaction occurs. We converted the interaction matrix into a distance matrix and used hierarchical clustering (hclust in R v.2.11.1) to construct a dendrogram detailing species similarity in trophic strategies [17], [25], [26]. We then measured total branch length of the dendrogram to quantify TD for each food web [17], [25]. In order to determine the best-fit dendrogram on which to base our measure of TD, we constructed dendrograms using Euclidean and Jaccard's distance measures and Unweighted Pair Group Method with Arithmetic Mean (UPGMA), Ward's, single, complete linkage methods. We selected Euclidean distance and UPGMA linkage method based on the cophenetic correlation [61], which measures how well the dendrogram represents the original similarity matrix. We standardized TD to range between zero and one [17].

### Measuring final TD, species richness, and species abundances

At the same time that we harvested the decomposed ants, we censused all the macro-invertebrates in each pitcher. Abundance of protozoa and rotifers was estimated as the average of five counts of 0.05-ml sub-samples. The protozoa *Cyclidium* and *Peranema* sporadically contaminated pitchers and unidentified microflagellates were found in nearly every pitcher. One pitcher contained two amphipods and another contained an unknown ciliate. The amphipods and unknown ciliate were not included in the sampling effect path model analysis. Final TD and species richness were calculated using the final community composition of each web including contaminant species (Table S3). Final TD and species richness were used in all analyses.

### Data analysis

We took a two step approach to analyzing the influence of species richness and TD on decomposition. First we assessed the univariate relationships between decomposition rate, final TD, and final species richness using regression models (lm in R v.2.11.1). For the second step in our analysis, we explored the mechanisms by which species richness and TD influence decomposition by using path analysis (sem in R v.2.11.1) to compare the variance-covariance structure (Table S4) of final TD, final species richness, ant decomposition, the abundance of each species, and initial ant mass in our *a priori* models (Fig. 1). We developed two classes of models. The first set of models included *sampling effect* models (Fig. 1a). In these models, species richness determines the abundance of a given species or TD, which in turn determines the rate of decomposition. We constructed 12 sampling effect models, one for each species (excluding amphipods and an unknown ciliate which were each present in only one pitcher). The second set of models included *food web* models (Fig. 1b–e). Food web models take into account known trophic interactions in the *Sarracenia* food web; protozoan abundances were summed in these models. *Complete food web* models included direct paths from mosquito larvae, rotifers, and copepods to bacterial decomposition and indirect effects via consuming protozoa (Fig.1c, e); *indirect food web* models contained only the indirect paths (Fig.1b, d). Every model was run with and without the initial ant mass directly connected to decomposition. Abundances were log_10_(*N*+1) transformed to normalize data and equalize variances.

We assessed the goodness-of-fit of each path model with the variance-covariance structure of the data using a Chi-square test (*P*-value >0.05 indicates a good fit) and the Comparative Fit Index (CFI; ref.[62]). A CFI >0.95 indicates a good fit between the hypothesized model and the observed data [63]. We compared models using the Bayesian Inference Criterion (BIC; ref. [64]) and considered model(s) with ΔBIC <6 as having strong support [65]. We calculated the proportion of variance (R^2^) in decomposition explained by each path model.

## Supporting Information

Figure S1 Relationship between realized trophic diversity (TD) and realized species richness for 70 pitcher plant food webs. Regression model (lm in R v.2.11.1) is significant at P<0.05.
(DOC)

Table S1 Experimental design table showing initial species richness, TD, and species composition of pitcher plant food webs. ID refers to the randomized position of the pitcher in the experiment. All food webs (including the “0” species webs) contained bacteria, ant carcasses, POM, and yeast. Columns on the right give species identities and initial abundances at the beginning of the experiment for species that do not reproduce in the pitcher.
(DOC)

Table S2 Interaction matrix of the *Sarracenia* food web used to calculate trophic diversity (TD) (31, 43, 44). “0”indicates no trophic interaction takes place and “1” indicates a trophic interaction.
(DOC)

Table S3 Comparison of initial and final trophic diversity (TD) and species richness.
(DOC)

Table S4 Covariance matrix used in path models.
(DOC)
