# Supplementary material for: Species Richness and Trophic Diversity Increase Decomposition in a Co-Evolved Food Web
Source: PLoS One. 2011 Jun 3;6(5):e20672. doi: 10.1371/journal.pone.0020672 (PMC3108618; doi:10.1371/journal.pone.0020672)
Supplement: Table S1 — Experimental design table showing initial species richness, TD, and species composition of pitcher plant food webs. ID refers to the randomized position of the pitcher in the experiment. All food webs (including the “0” species webs) contained bacteria, ant carcasses, POM, and yeast. Columns on the right give species identities and initial abundances at the beginning of the experiment for species that do not reproduce in the pitcher. (DOC) [file pone.0020672.s002.doc]

**Table S1.**

| **Id.** | **Sp. Rich** | **Initial TD** | **Species composition** | | | | | | | | | | | | | **Sp. Id.** | **Sp. Name** | **Abundance** |
| --- | --- | --- | --- | --- | --- | --- | --- | --- | --- | --- | --- | --- | --- | --- | --- | --- | --- | --- |
| 2 | 0 | 4.25 | 10 | 11 | 12 | 13 |  |  |  |  |  |  |  |  |  | 1 | Midge | 10 |
| 8 | 0 | 4.25 | 10 | 11 | 12 | 13 |  |  |  |  |  |  |  |  |  | 2 | *Paramecium* | 1 |
| 10 | 0 | 4.25 | 10 | 11 | 12 | 13 |  |  |  |  |  |  |  |  |  | 3 | *Haltera* | - |
| 13 | 0 | 4.25 | 10 | 11 | 12 | 13 |  |  |  |  |  |  |  |  |  | 4 | *Bodo* | - |
| 53 | 0 | 4.25 | 10 | 11 | 12 | 13 |  |  |  |  |  |  |  |  |  | 5 | *Colpidium* | - |
| 62 | 0 | 4.25 | 10 | 11 | 12 | 13 |  |  |  |  |  |  |  |  |  | 6 | mite | 10 |
| 69 | 0 | 4.25 | 10 | 11 | 12 | 13 |  |  |  |  |  |  |  |  |  | 7 | rotifer | - |
| 54 | 1 | 4.86 | 5 | 10 | 11 | 12 | 13 |  |  |  |  |  |  |  |  | 8 | copepod | 2 |
| 9 | 1 | 4.87 | 9 | 10 | 11 | 12 | 13 |  |  |  |  |  |  |  |  | 9 | mosquito | 5 |
| 15 | 1 | 4.87 | 3 | 10 | 11 | 12 | 13 |  |  |  |  |  |  |  |  | 10 | Bacteria | - |
| 20 | 1 | 4.87 | 8 | 10 | 11 | 12 | 13 |  |  |  |  |  |  |  |  | 11 | Ant carcass | - |
| 1 | 1 | 6.30 | 1 | 10 | 11 | 12 | 13 |  |  |  |  |  |  |  |  | 12 | POM | - |
| 37 | 1 | 7.08 | 6 | 10 | 11 | 12 | 13 |  |  |  |  |  |  |  |  | 13 | Yeast | - |
| 57 | 1 | 7.50 | 7 | 10 | 11 | 12 | 13 |  |  |  |  |  |  |  |  |  |  |  |
| 44 | 2 | 5.38 | 8 | 9 | 10 | 11 | 12 | 13 |  |  |  |  |  |  |  |  |  |  |
| 33 | 2 | 7.08 | 1 | 4 | 10 | 11 | 12 | 13 |  |  |  |  |  |  |  |  |  |  |
| 49 | 2 | 7.53 | 6 | 8 | 10 | 11 | 12 | 13 |  |  |  |  |  |  |  |  |  |  |
| 7 | 2 | 8.11 | 3 | 7 | 10 | 11 | 12 | 13 |  |  |  |  |  |  |  |  |  |  |
| 34 | 2 | 8.94 | 1 | 6 | 10 | 11 | 12 | 13 |  |  |  |  |  |  |  |  |  |  |
| 27 | 2 | 9.31 | 6 | 7 | 10 | 11 | 12 | 13 |  |  |  |  |  |  |  |  |  |  |
| 30 | 2 | 9.43 | 1 | 7 | 10 | 11 | 12 | 13 |  |  |  |  |  |  |  |  |  |  |
| 61 | 3 | 5.83 | 3 | 4 | 5 | 10 | 11 | 12 | 13 |  |  |  |  |  |  |  |  |  |
| 22 | 3 | 7.95 | 2 | 3 | 6 | 10 | 11 | 12 | 13 |  |  |  |  |  |  |  |  |  |
| 19 | 3 | 8.35 | 3 | 4 | 8 | 10 | 11 | 12 | 13 |  |  |  |  |  |  |  |  |  |
| 31 | 3 | 8.56 | 2 | 3 | 7 | 10 | 11 | 12 | 13 |  |  |  |  |  |  |  |  |  |
| 46 | 3 | 10.31 | 1 | 4 | 7 | 10 | 11 | 12 | 13 |  |  |  |  |  |  |  |  |  |
| 43 | 3 | 10.67 | 5 | 6 | 9 | 10 | 11 | 12 | 13 |  |  |  |  |  |  |  |  |  |
| 3 | 3 | 11.27 | 1 | 6 | 7 | 10 | 11 | 12 | 13 |  |  |  |  |  |  |  |  |  |
| 35 | 4 | 6.24 | 2 | 3 | 4 | 5 | 10 | 11 | 12 | 13 |  |  |  |  |  |  |  |  |
| 17 | 4 | 8.34 | 3 | 4 | 5 | 6 | 10 | 11 | 12 | 13 |  |  |  |  |  |  |  |  |
| 51 | 4 | 8.98 | 2 | 4 | 5 | 7 | 10 | 11 | 12 | 13 |  |  |  |  |  |  |  |  |
| 36 | 4 | 10.70 | 1 | 2 | 4 | 7 | 10 | 11 | 12 | 13 |  |  |  |  |  |  |  |  |
| 28 | 4 | 11.00 | 1 | 2 | 3 | 9 | 10 | 11 | 12 | 13 |  |  |  |  |  |  |  |  |
| 55 | 4 | 11.49 | 3 | 5 | 6 | 9 | 10 | 11 | 12 | 13 |  |  |  |  |  |  |  |  |
| 42 | 4 | 13.57 | 4 | 6 | 7 | 8 | 10 | 11 | 12 | 13 |  |  |  |  |  |  |  |  |
| 25 | 5 | 8.24 | 2 | 3 | 4 | 5 | 7 | 10 | 11 | 12 | 13 |  |  |  |  |  |  |  |
| 11 | 5 | 9.58 | 2 | 3 | 5 | 8 | 9 | 10 | 11 | 12 | 13 |  |  |  |  |  |  |  |
| 52 | 5 | 10.75 | 1 | 2 | 4 | 5 | 6 | 10 | 11 | 12 | 13 |  |  |  |  |  |  |  |
| 58 | 5 | 12.05 | 1 | 3 | 6 | 7 | 8 | 10 | 11 | 12 | 13 |  |  |  |  |  |  |  |
| 4 | 5 | 13.47 | 1 | 4 | 5 | 6 | 9 | 10 | 11 | 12 | 13 |  |  |  |  |  |  |  |
| 64 | 5 | 14.01 | 5 | 6 | 7 | 8 | 9 | 10 | 11 | 12 | 13 |  |  |  |  |  |  |  |
| 63 | 5 | 15.89 | 1 | 3 | 6 | 7 | 8 | 10 | 11 | 12 | 13 |  |  |  |  |  |  |  |
| 14 | 6 | 9.66 | 2 | 3 | 4 | 5 | 8 | 9 | 10 | 11 | 12 | 13 |  |  |  |  |  |  |
| 26 | 6 | 11.49 | 1 | 2 | 3 | 4 | 5 | 7 | 10 | 11 | 12 | 13 |  |  |  |  |  |  |
| 29 | 6 | 11.77 | 1 | 2 | 3 | 4 | 5 | 9 | 10 | 11 | 12 | 13 |  |  |  |  |  |  |
| 59 | 6 | 12.80 | 2 | 3 | 4 | 5 | 6 | 8 | 10 | 11 | 12 | 13 |  |  |  |  |  |  |
| 56 | 6 | 14.61 | 1 | 2 | 3 | 4 | 7 | 9 | 10 | 11 | 12 | 13 |  |  |  |  |  |  |
| 70 | 6 | 14.91 | 2 | 4 | 6 | 7 | 8 | 9 | 10 | 11 | 12 | 13 |  |  |  |  |  |  |
| 60 | 6 | 16.67 | 1 | 2 | 4 | 6 | 7 | 9 | 10 | 11 | 12 | 13 |  |  |  |  |  |  |
| 40 | 7 | 13.12 | 2 | 3 | 4 | 5 | 6 | 8 | 9 | 10 | 11 | 12 | 13 |  |  |  |  |  |
| 41 | 7 | 13.19 | 2 | 3 | 4 | 5 | 7 | 8 | 9 | 10 | 11 | 12 | 13 |  |  |  |  |  |
| 47 | 7 | 14.15 | 1 | 2 | 3 | 4 | 5 | 6 | 9 | 10 | 11 | 12 | 13 |  |  |  |  |  |
| 32 | 7 | 15.04 | 1 | 2 | 3 | 4 | 5 | 7 | 9 | 10 | 11 | 12 | 13 |  |  |  |  |  |
| 65 | 7 | 15.44 | 3 | 4 | 5 | 6 | 7 | 8 | 9 | 10 | 11 | 12 | 13 |  |  |  |  |  |
| 24 | 7 | 16.87 | 1 | 2 | 3 | 4 | 6 | 7 | 9 | 10 | 11 | 12 | 13 |  |  |  |  |  |
| 12 | 7 | 17.52 | 1 | 2 | 3 | 6 | 7 | 8 | 9 | 10 | 11 | 12 | 13 |  |  |  |  |  |
| 50 | 8 | 15.50 | 1 | 2 | 3 | 4 | 5 | 7 | 8 | 9 | 10 | 11 | 12 | 13 |  |  |  |  |
| 18 | 8 | 15.65 | 2 | 3 | 4 | 5 | 6 | 7 | 8 | 9 | 10 | 11 | 12 | 13 |  |  |  |  |
| 5 | 8 | 17.24 | 1 | 2 | 3 | 4 | 5 | 6 | 7 | 9 | 10 | 11 | 12 | 13 |  |  |  |  |
| 68 | 8 | 17.24 | 1 | 2 | 3 | 4 | 5 | 6 | 7 | 8 | 10 | 11 | 12 | 13 |  |  |  |  |
| 16 | 8 | 18.02 | 1 | 3 | 4 | 5 | 6 | 7 | 8 | 9 | 10 | 11 | 12 | 13 |  |  |  |  |
| 23 | 8 | 18.02 | 1 | 2 | 3 | 4 | 6 | 7 | 8 | 9 | 10 | 11 | 12 | 13 |  |  |  |  |
| 39 | 8 | 18.17 | 1 | 2 | 3 | 4 | 5 | 6 | 8 | 9 | 10 | 11 | 12 | 13 |  |  |  |  |
| 38 | 9 | 15.24 | 1 | 2 | 3 | 4 | 5 | 6 | 7 | 8 | 9 | 10 | 11 | 12 | 13 |  |  |  |
| 45 | 9 | 18.17 | 1 | 2 | 3 | 4 | 5 | 6 | 7 | 8 | 9 | 10 | 11 | 12 | 13 |  |  |  |
| 48 | 9 | 18.17 | 1 | 2 | 3 | 4 | 5 | 6 | 7 | 8 | 9 | 10 | 11 | 12 | 13 |  |  |  |
| 66 | 9 | 18.17 | 1 | 2 | 3 | 4 | 5 | 6 | 7 | 8 | 9 | 10 | 11 | 12 | 13 |  |  |  |
| 67 | 9 | 18.17 | 1 | 2 | 3 | 4 | 5 | 6 | 7 | 8 | 9 | 10 | 11 | 12 | 13 |  |  |  |
| 6 | 9 | 18.17 | 1 | 2 | 3 | 4 | 5 | 6 | 7 | 8 | 9 | 10 | 11 | 12 | 13 |  |  |  |
| 21 | 9 | 18.17 | 1 | 2 | 3 | 4 | 5 | 6 | 7 | 8 | 9 | 10 | 11 | 12 | 13 |  |  |  |
