# Supplementary material for: Species Richness and Trophic Diversity Increase Decomposition in a Co-Evolved Food Web
Source: PLoS One. 2011 Jun 3;6(5):e20672. doi: 10.1371/journal.pone.0020672 (PMC3108618; doi:10.1371/journal.pone.0020672)
Supplement: Table S3 — Comparison of initial and final trophic diversity (TD) and species richness. (DOC) [file pone.0020672.s004.doc]

**Table S3**

| **Block #** | **Id #** | **Initial Sp. Richness** | **Final Sp. Richness** | **Initial TD** | **Final TD** |
| --- | --- | --- | --- | --- | --- |
| 1 | 2 | 0 | 1 | 4.25 | 4.86 |
| 2 | 8 | 0 | 3 | 4.25 | 7.95 |
| 2 | 10 | 0 | 1 | 4.25 | 4.86 |
| 2 | 13 | 0 | 3 | 4.25 | 7.53 |
| 8 | 53 | 0 | 2 | 4.25 | 5.38 |
| 9 | 62 | 0 | 3 | 4.25 | 8.35 |
| 10 | 69 | 0 | 1 | 4.25 | 4.87 |
| 1 | 1 | 1 | 2 | 6.30 | 7.08 |
| 2 | 9 | 1 | 2 | 4.87 | 7.93 |
| 3 | 15 | 1 | 4 | 4.87 | 8.34 |
| 3 | 20 | 1 | 4 | 4.87 | 11.68 |
| 6 | 37 | 1 | 3 | 7.08 | 7.95 |
| 8 | 54 | 1 | 2 | 4.86 | 5.38 |
| 9 | 57 | 1 | 3 | 7.50 | 8.57 |
| 1 | 7 | 2 | 3 | 8.11 | 10.31 |
| 4 | 27 | 2 | 5 | 9.31 | 13.25 |
| 5 | 30 | 2 | 3 | 9.43 | 10.31 |
| 5 | 33 | 2 | 2 | 7.08 | 7.08 |
| 5 | 34 | 2 | 5 | 8.94 | 11.29 |
| 7 | 44 | 2 | 4 | 5.38 | 9 |
| 7 | 49 | 2 | 4 | 7.53 | 11.49 |
| 1 | 3 | 3 | 4 | 11.27 | 13.23 |
| 3 | 19 | 3 | 4 | 8.35 | 8.35 |
| 4 | 22 | 3 | 3 | 7.95 | 7.95 |
| 5 | 31 | 3 | 4 | 8.56 | 11.34 |
| 7 | 43 | 3 | 5 | 10.67 | 13.47 |
| 7 | 46 | 3 | 5 | 10.31 | 13.54 |
| 9 | 61 | 3 | 3 | 5.83 | 5.83 |
| 3 | 17 | 4 | 3 | 8.34 | 7.95 |
| 4 | 28 | 4 | 5 | 11.00 | 11.68 |
| 5 | 35 | 4 | 2 | 6.24 | 5.4 |
| 6 | 36 | 4 | 3 | 10.70 | 10.31 |
| 6 | 42 | 4 | 6 | 13.57 | 16.67 |
| 8 | 51 | 4 | 4 | 8.98 | 8.98 |
| 8 | 55 | 4 | 4 | 11.49 | 11.49 |
| 1 | 4 | 5 | 5 | 13.47 | 16.67 |
| 2 | 11 | 5 | 5 | 9.58 | 15.96 |
| 4 | 25 | 5 | 4 | 8.24 | 13.27 |
| 8 | 52 | 5 | 5 | 10.75 | 15.96 |
| 9 | 58 | 5 | 7 | 12.05 | 16.54 |
| 9 | 63 | 5 | 8 | 15.89 | 18.72 |
| 10 | 64 | 5 | 7 | 14.01 | 16.88 |
| 2 | 14 | 6 | 2 | 9.66 | 7.53 |
| 4 | 26 | 6 | 3 | 11.49 | 10.31 |
| 5 | 29 | 6 | 5 | 11.77 | 11.68 |
| 8 | 56 | 6 | 5 | 14.61 | 13.29 |
| 9 | 59 | 6 | 2 | 12.80 | 7.53 |
| 9 | 60 | 6 | 6 | 16.67 | 16.67 |
| 10 | 70 | 6 | 6 | 14.91 | 14.49 |
| 2 | 12 | 7 | 4 | 17.52 | 15.95 |
| 4 | 24 | 7 | 6 | 16.87 | 14.61 |
| 5 | 32 | 7 | 5 | 15.04 | 14.2 |
| 6 | 40 | 7 | 3 | 13.12 | 10.67 |
| 6 | 41 | 7 | 8 | 13.19 | 15.3 |
| 7 | 47 | 7 | 7 | 14.15 | 14.15 |
| 10 | 65 | 7 | 7 | 15.44 | 14.91 |
| 1 | 5 | 8 | 9 | 17.24 | 17.24 |
| 3 | 16 | 8 | 6 | 18.02 | 14.86 |
| 3 | 18 | 8 | 8 | 15.65 | 16.63 |
| 4 | 23 | 8 | 7 | 18.02 | 18.23 |
| 6 | 39 | 8 | 5 | 18.17 | 13.47 |
| 8 | 50 | 8 | 8 | 15.50 | 17.61 |
| 10 | 68 | 8 | 5 | 17.24 | 15.4 |
| 1 | 6 | 9 | 6 | 18.17 | 14.61 |
| 3 | 21 | 9 | 9 | 18.17 | 19.5 |
| 6 | 38 | 9 | 6 | 15.24 | 14.08 |
| 7 | 45 | 9 | 7 | 18.17 | 16.54 |
| 7 | 48 | 9 | 6 | 18.17 | 16.45 |
| 10 | 66 | 9 | 6 | 18.17 | 16.67 |
| 10 | 67 | 9 | 5 | 18.17 | 15.96 |
