# Supplementary material for: Species Richness and Trophic Diversity Increase Decomposition in a Co-Evolved Food Web
Source: PLoS One. 2011 Jun 3;6(5):e20672. doi: 10.1371/journal.pone.0020672 (PMC3108618; doi:10.1371/journal.pone.0020672)
Supplement: Table S4 — Covariance matrix used in path models. (DOC) [file pone.0020672.s005.doc]

|  | Sp. Rich | AntMass | TD | Decomp | Prot. combined | Midge | Mite | Copepod | Mosquito | Rotifer | P..bursaria | Haltera | Bodo | Colpidium | Cyclidium | Perenema | Micro flags |
| --- | --- | --- | --- | --- | --- | --- | --- | --- | --- | --- | --- | --- | --- | --- | --- | --- | --- |
| Sp. Rich | 3.90E+00 | -8.65E-04 | 3.72E-01 | 1.38E-01 | -1.35E-02 | 4.00E-01 | 3.26E-01 | 9.09E-02 | 2.88E-01 | 8.92E-02 | 5.31E-01 | 1.39E-01 | 9.68E-01 | 3.38E-01 | 1.44E-01 | 3.87E-01 | -1.41E-01 |
| AntMass | -8.65E-04 | 7.42E-05 | -4.79E-05 | -6.41E-04 | -3.32E-05 | -1.95E-04 | -7.60E-04 | -3.18E-05 | -2.64E-04 | -2.44E-04 | 3.68E-04 | -2.44E-04 | -1.52E-03 | 8.64E-04 | 4.19E-04 | 5.51E-04 | 1.97E-04 |
| TD | 3.72E-01 | -4.79E-05 | 4.34E-02 | 1.81E-02 | -1.73E-02 | 5.60E-02 | 4.18E-02 | 6.89E-03 | 3.30E-02 | 1.43E-02 | 3.29E-02 | 1.29E-02 | 3.05E-02 | 2.51E-02 | 9.91E-03 | 3.13E-02 | -3.01E-02 |
| Decomp | 1.38E-01 | -6.41E-04 | 1.81E-02 | 4.25E-02 | -3.77E-02 | 4.83E-02 | 1.27E-02 | -1.97E-05 | 1.12E-02 | 7.12E-03 | 5.37E-03 | 8.93E-03 | -3.15E-03 | 9.20E-03 | -2.65E-03 | 1.11E-02 | -4.83E-02 |
| Prot. combined | -1.35E-02 | -3.32E-05 | -1.73E-02 | -3.77E-02 | 6.63E-01 | -1.49E-02 | -1.26E-01 | 1.57E-02 | -8.35E-02 | 4.56E-02 | 6.04E-02 | -1.88E-02 | 4.96E-01 | 4.52E-03 | -2.81E-02 | 1.80E-01 | 7.82E-01 |
| Midge | 4.00E-01 | -1.95E-04 | 5.60E-02 | 4.83E-02 | -1.49E-02 | 2.20E-01 | -1.23E-02 | 4.99E-04 | 3.36E-02 | 1.70E-02 | 3.50E-03 | 2.30E-02 | -1.29E-01 | 9.53E-03 | 1.77E-02 | 6.77E-03 | -2.75E-02 |
| Mite | 3.26E-01 | -7.60E-04 | 4.18E-02 | 1.27E-02 | -1.26E-01 | -1.23E-02 | 2.82E-01 | -4.54E-03 | 3.64E-02 | -5.11E-03 | -1.19E-02 | 3.11E-02 | -3.15E-02 | -7.66E-03 | 3.29E-02 | -3.46E-02 | -1.55E-01 |
| Copepod | 9.09E-02 | -3.18E-05 | 6.89E-03 | -1.97E-05 | 1.57E-02 | 4.99E-04 | -4.54E-03 | 1.74E-02 | 8.97E-04 | 7.19E-04 | 1.57E-02 | 3.55E-03 | 5.29E-02 | -4.95E-03 | 2.93E-03 | 2.26E-02 | 1.03E-02 |
| Mosquito | 2.88E-01 | -2.64E-04 | 3.30E-02 | 1.12E-02 | -8.35E-02 | 3.36E-02 | 3.64E-02 | 8.97E-04 | 1.11E-01 | -2.75E-02 | 3.47E-02 | -1.61E-02 | 4.65E-02 | 2.84E-02 | -4.99E-03 | -5.24E-02 | -1.09E-01 |
| Rotifer | 8.92E-02 | -2.44E-04 | 1.43E-02 | 7.12E-03 | 4.56E-02 | 1.70E-02 | -5.11E-03 | 7.19E-04 | -2.75E-02 | 1.03E-01 | 2.47E-02 | 3.41E-03 | -6.48E-02 | -3.26E-03 | 1.84E-03 | 3.72E-02 | 5.96E-02 |
| P..bursaria | 5.31E-01 | 3.68E-04 | 3.29E-02 | 5.37E-03 | 6.04E-02 | 3.50E-03 | -1.19E-02 | 1.57E-02 | 3.47E-02 | 2.47E-02 | 2.54E-01 | -1.54E-02 | 1.87E-01 | 6.68E-02 | 1.02E-02 | 7.29E-02 | 7.05E-02 |
| Haltera | 1.39E-01 | -2.44E-04 | 1.29E-02 | 8.93E-03 | -1.88E-02 | 2.30E-02 | 3.11E-02 | 3.55E-03 | -1.61E-02 | 3.41E-03 | -1.54E-02 | 1.05E-01 | -8.74E-03 | -1.11E-02 | 3.37E-02 | 2.13E-02 | -2.49E-02 |
| Bodo | 9.68E-01 | -1.52E-03 | 3.05E-02 | -3.15E-03 | 4.96E-01 | -1.29E-01 | -3.15E-02 | 5.29E-02 | 4.65E-02 | -6.48E-02 | 1.87E-01 | -8.74E-03 | 1.91E+00 | 1.32E-01 | -6.58E-02 | 3.01E-01 | 4.18E-01 |
| Colpidium | 3.38E-01 | 8.64E-04 | 2.51E-02 | 9.20E-03 | 4.52E-03 | 9.53E-03 | -7.66E-03 | -4.95E-03 | 2.84E-02 | -3.26E-03 | 6.68E-02 | -1.11E-02 | 1.32E-01 | 2.46E-01 | -8.74E-03 | 8.51E-02 | -9.59E-03 |
| Cyclidium | 1.44E-01 | 4.19E-04 | 9.91E-03 | -2.65E-03 | -2.81E-02 | 1.77E-02 | 3.29E-02 | 2.93E-03 | -4.99E-03 | 1.84E-03 | 1.02E-02 | 3.37E-02 | -6.58E-02 | -8.74E-03 | 9.51E-02 | -2.34E-02 | -2.58E-02 |
| Perenema | 3.87E-01 | 5.51E-04 | 3.13E-02 | 1.11E-02 | 1.80E-01 | 6.77E-03 | -3.46E-02 | 2.26E-02 | -5.24E-02 | 3.72E-02 | 7.29E-02 | 2.13E-02 | 3.01E-01 | 8.51E-02 | -2.34E-02 | 3.66E-01 | 1.98E-01 |
| Micro flags | -1.41E-01 | 1.97E-04 | -3.01E-02 | -4.83E-02 | 7.82E-01 | -2.75E-02 | -1.55E-01 | 1.03E-02 | -1.09E-01 | 5.96E-02 | 7.05E-02 | -2.49E-02 | 4.18E-01 | -9.59E-03 | -2.58E-02 | 1.98E-01 | 1.04E+00 |

**Table S4**
